# Extending the sufficient component cause model to describe the Stable Unit Treatment Value Assumption (SUTVA)

**DOI:** 10.1186/1742-5573-9-3

**Published:** 2012-04-03

**Authors:** Sharon Schwartz, Nicolle M Gatto, Ulka B Campbell

**Affiliations:** 1 Department of Epidemiology, Mailman School of Public Health, Columbia University, 722 West 168 Street, NY, New York 10032, USA; 2 Department of Epidemiology, Columbia University Mailman School of Public Health, NY, New York, USA; 3 Epidemiology, Worldwide Safety Strategy, Pfizer Inc, NY, New York, USA

**Keywords:** Causal inference, Consistency assumption, Counterfactuals, Stability assumption, SUTVA

## Abstract

Causal inference requires an understanding of the conditions under which association equals causation. The exchangeability or no confounding assumption is well known and well understood as central to this task. More recently the epidemiologic literature has described additional assumptions related to the stability of causal effects. In this paper we extend the Sufficient Component Cause Model to represent one expression of this stability assumption--the Stable Unit Treatment Value Assumption. Approaching SUTVA from an SCC model helps clarify what SUTVA is and reinforces the connections between interaction and SUTVA.

## Introduction

The potential outcomes approach is becoming the standard for causal inference in epidemiology [1]. By providing a precise definition of a causal effect, this approach clarifies the assumptions necessary for the 'associational smoke' of our data to indicate 'causational fire', to use Holland's evocative metaphor [2]. One key requirement, which has received much attention in epidemiology is the "no confounding" or exchangeability assumption. More recently, epidemiologists have argued that this assumption, even in the absence of measurement error, is not sufficient.

For example, reflecting on their seminal paper "Identifiability, Exchangeability and Confounding",[3] Greenland and Robins noted as a shortcoming that this paper "did not emphasize the importance of limiting the exposure × to a potentially changeable condition". That is, in describing the conditions necessary for association to equal causation, they neglected to invoke Holland's mantra "no causation without manipulation" [2] or what might be referred to as a manipulability assumption. Related expressions include the consistency [4], "no treatment variation" [5], and "no interference" [5] or "no interaction between subjects" [6] assumptions. An early formulation, given by Rubin, [7,8] is the Stable Unit Treatment Value assumption (SUTVA).

What unites these assumptions is that they are required for well-defined causal questions from a potential outcomes perspective -- causal questions that can be posed as comparisons between "two or more well-defined interventions" [4] . Recent discussions by Cole and Frangakis,[9] VanderWeele, [5] Pearl, [10] and Peterson [11] clarified the role of the consistency assumption, provided mathematical notation for it and examined it using causal graphs. Hernan and VanderWeele conclude from this work that "interference between units" and what they refer to as "versions of compound treatments" limit the transportability of causal effects from one population to another just as interaction does [12].

These assumptions have been examined from the perspective of potential outcomes [11,12] and Directed Acyclic Graphs. In this paper, we show how the Sufficient Component Cause model can be extended to represent Rubin's expression of the no interference between units and the no compound versions of treatment assumptions -- the Stable Unit Treatment Value Assumption (SUTVA) [7,8]. In so doing we hope to extend the utility of the Sufficient Component Cause model. We also think that examining SUTVA through an SCC lens crystallizes the kinship between interaction and SUTVA, reinforcing why SUTVA is necessary for transportability. Just as the SCC model allows us to see the ubiquity of interactions, it also allows us to see the ubiquity of SUTVA violations.

To lay the groundwork, we first provide a brief overview of the potential outcomes approach.

### The potential outcomes model

#### Definition of a causal effect

The guiding metaphor of the potential outcomes frame is the randomized controlled trial. From this perspective, causal effects are defined as hypothetical intervention effects. A causal effect is the difference between the outcomes (e.g., disease or not) that would arise for an individual by the end of the observation period under two different exposure conditions. In considering a disease outcome, each individual is conceptualized as having a fixed potential outcome for the disease by the end of the observation period under each exposure condition, regardless of their actual exposure status. Therefore, when comparing two exposure conditions (e.g., exposed and unexposed), there are four possible sets of potential outcomes (i.e., response types) for each individual [13]. There is a causal effect of the exposure on the outcome if and only if an individual's potential outcomes by the end of the study period would be different under the two exposure conditions.

Because a causal effect is defined as the difference in the outcome in the same individual (or more generally the same target or unit) at the same time but with different exposure experiences, a causal effect can never be seen [2]. To estimate causal effects from observations, epidemiologists use substitutes for the missing data. These substitutes can provide the correct answer if the potential outcomes of the substitutes are the same as the potential outcomes of the people for whom you want to estimate a causal effect. While we generally cannot hope to find substitutes for the potential outcomes of a particular individual, we can hope to find substitutes that represent the distribution of potential outcomes among a group of individuals [14]. This is referred to as the exchangeability or no confounding assumption [13] and is well known, well accepted and well studied, in epidemiology.

But an additional assumption is necessary to estimate the precise causal effect defined from a potential outcomes perspective - SUTVA, the Stable Unit Treatment Value Assumption [8].

### What is SUTVA?

The Stable Unit Treatment Value Assumption, as developed by Rubin [7,8], is the assumption that each individual has only one potential outcome under each exposure condition. This is necessary to ensure that the causal effect for each individual is stable. This stability assumption has two elements:

1. that the exposure has the same effect on an individual regardless of how the individual came to be exposed, and

2. that the effect of the exposure on an individual is independent of the exposure of other individuals

Within epidemiology, the first aspect of SUTVA is sometimes referred to as the consistency assumption [4] and the treatment variation irrelevance assumption [5]. In epidemiology, the term SUTVA is often reserved for the second assumption[15] although Cole and Frangakis connect the first assumption with the consistency assumption [9].

Violation of either aspect of SUTVA creates unstable estimates of the causal effect. By unstable we mean that there is no unique potential outcome for each individual under each exposure condition. In general, the instability arises because there are multiple "versions of treatment". Although the treatment was defined as a single construct, it really represents different versions of treatments that were not recognized and delineated. Each version of the treatment may influence a particular individual in a different way. Thus an individual may have more than one potential outcome for what is considered in the study to be a single treatment condition. These different versions of treatment arise from ambiguities in the measurement or operationalization of the treatment (violation of the first aspect of SUTVA) or from effects of the treatments received by others (violation of the second aspect of SUTVA).

We now build a Sufficient Component Cause Model [16] and define corresponding potential outcomes and response types [13] when exchangeability and SUTVA hold. We then extend this model to depict violations of SUTVA.

### SCC model and response types for a single dichotomous exposure and outcome with no SUTVA violation

**Figure 1:**
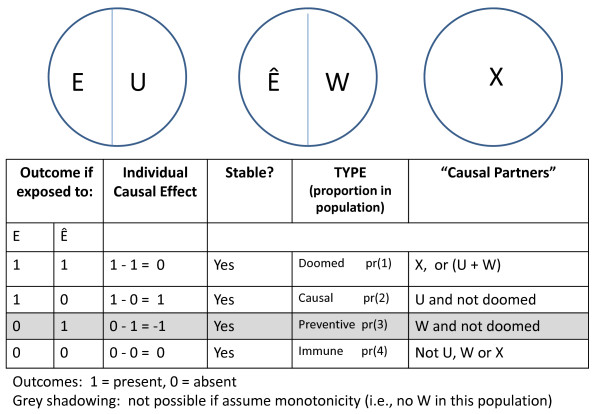
**Simple SCC model and corresponding response types**.

Figure 1 depicts a minimal Sufficient Component Cause model that describes the effects of an exposure on an outcome. We assume that there are no SUTVA violations in this example. The exposure is precisely defined and therefore we can easily imagine an intervention that would remove the exposure from the population. In the first sufficient cause, this precisely defined exposure works with its "causal partners" (denoted by U) to cause disease. By "causal partners", we mean the other component causes that activate or allow the exposure to cause the disease. A second sufficient cause indicates that the exposure can be preventive (denoted by Ê, the absence of exposure) in the context of other causal partners (W). Individuals can also get the disease from mechanisms that do not include the exposure under study; these other unspecified mechanisms are denoted by X.

The table at the bottom of Figure 1 displays the sets of potential outcomes implied by this simple Sufficient Component Cause model. If we assume monotonicity (i.e., in this context, that E can be causal but not preventive), there are no individuals in this population with the set of potential outcomes shown in the grey row. This would happen, for example, if no one in this population were exposed to W.

The causal effect for each individual is defined by the difference between the potential outcomes of the individual under the two different exposure conditions. For example, the exposure would have no causal effect for individuals with the sets of potential outcomes depicted in the first and the last rows; the difference between the two potential outcomes for these individuals is 0. For individuals in the first row, labeled as "doomed" types [12], the exposure has no effect because whether or not they are exposed, they get the disease. Their potential outcome under both exposed and unexposed conditions is 1 and thus the difference between their potential outcomes under exposure and non-exposure (i.e., 1-1) is 0.

For individuals in the last row, labeled as "immune" types, the exposure has no effect because whether or not they are exposed, they do not get the disease. Their potential outcome under both exposed and unexposed conditions is 0. Under monotonicity, the exposure only has an effect on individuals in the second row, labeled the "causal" types. Their potential outcomes under exposed and unexposed conditions differ (1-0 = 1) and thus the exposure has a causal effect for them. Since there are no SUTVA violations, the causal effect is stable for all individuals.

The causal effect for a population would be a comparison between the potential outcomes (i.e., risk of disease) if everyone were exposed compared with the risk of disease if everyone were unexposed. This represents the average of the causal effects for the individuals in the population. This average causal effect can be expressed in terms of the population proportions of the different response types.

The doomed and causal types are those who develop disease under exposure and the doomed and preventive types are those who develop disease under non-exposure. Thus, the causal effect can be expressed as a contrast between the sum of the doomed and causal proportions and the sum of the doomed and preventive proportions. Under monotonicity, the average causal risk difference is represented by the proportion of causal types in the population, pr(2). When monotonicity does not hold the average causal risk difference is represented by the proportion of causal types, pr(2), minus the proportion of preventive types, pr(3).

Note that in estimating causal effects, we always "give priority to the doomed". That is, if an individual has both U_1 _and × by the end of the study period the exposure has no causal effect for the individual even if she actually got the disease from the exposure. The causal effect is defined as the difference in potential outcomes at the end of the study period and if an individual had × by that time, there would be no difference in the individual's potential outcomes by the end of the study period [17]. Nonetheless, if the individual had U_1 _before X, the exposure actually would have caused her disease despite having no causal effect on the disease. That is, epidemiologic methods generally estimate only excess (i.e., end of study) but not etiologic (i.e., actual) effects [18].

Of course we cannot "see" the causal effect in a population because we do not know the distribution of types. However, if we imagine randomly assigning exposure to individuals in this population, then on average the distribution of types in the exposed and unexposed groups will be the same and the average causal effect in this population will be identifiable. In observational studies too, the average causal effect will be identifiable if the distribution of types is the same in the exposed and unexposed groups -- i.e., if the two groups are exchangeable. This provides a clear meaning for the exchangeability or no confounding assumption [13].

To ease the extension of this model into violations of SUTVA we will use the following example. Suppose we are interested in estimating the causal effect of bicycle riding (defined as riding 30 miles a week) on weight loss (defined as losing 5 pounds or more) by the end of the study period. We can imagine randomly assigning each individual in an infinitely large population to a "bicycle riding" or "no bicycle riding" condition and noting if they lose weight by the end of the study period. If this were an idealized randomized controlled trial (i.e., full compliance - everyone rides either 30 miles a week or not all, no loss to follow-up, infinite sample size), the average causal effect in this population would be a contrast between the proportions of individuals who lose weight among the bicycle riding and no bicycle riding conditions.

We can also express these sets of potential outcomes from the perspective of a sufficient component cause model. We can conceptualize the Sufficient Component Cause model in Figure 1 as depicting the sufficient causes for weight loss from the perspective of this intervention. E now represents the bicycle riding condition, and Ê the no bicycle riding condition. Some individuals in this population would lose weight if they were exposed --those who would have the causal partners of the exposure, U, by the end of the study period. Other individuals, those who would have × by the end of the study period, would lose weight with or without the exposure. Some individuals, those with W, will only lose weight if they remain unexposed. Individuals without U, X, and W will not lose weight, regardless of their exposure status.

### Sufficient component cause model and response types to depict SUTVA

To estimate a precise causal effect of bicycle riding on weight loss in this population, it is assumed that each individual has one potential outcome under the exposed condition and one potential outcome under the unexposed condition. SUTVA is an articulation of this assumption and the ways in which it can be violated. SUTVA is violated if the random assignment of the individual to the same treatment condition at the same moment in time could result in different outcomes. How could this happen? As we noted above, there are two general ways in which SUTVA could be violated. We discuss them in turn below.

### Violation of SUTVA through different versions of treatment

**Figure 2:**
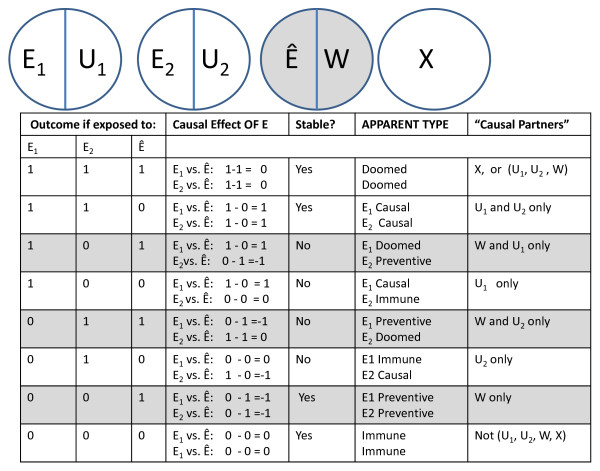
**Simple SCC model and corresponding response types representing different versions of treatment**. Grey shadowing indicates not present under monotonicity

SUTVA could be violated if in operationalizing the exposure, there are really two different versions that are being randomly assigned. In our example, suppose everyone assigned to bicycle riding is assigned to ride 30 miles a week on a local bike path. Unbeknownst to the researcher, some of the rides take place on a part of the bike path with a hilly terrain and other rides take place on a flat terrain. If the energy expenditure of the two different versions of bike riding would have different effects for the same individual, there are three (hilly ride, flat ride, or no ride) rather than two potential outcomes for the set of exposure and non-exposure conditions. For some individuals, their response type is not stable because they are a different "type" depending on which version of exposure they received. Here, unrepresented versions of the exposure would violate SUTVA. Figure 2 depicts a Sufficient Component Cause model and corresponding response types to represent a violation of SUTVA from an operationalization that leads to different, unrepresented versions of the treatment.

The variable that we considered a single unified exposure in our imagined randomized control trial, bicycle riding, (E) is actually two different exposures (E_1 _= flat rides and E_2 _= hilly rides), each of which has a unique effect on some individuals in the population. A precise causal effect in this population, that is the effect of assigning everyone to ride 30 miles a week compared with assigning everyone to not ride, would not be precisely definable in this population. The causal effect would depend on: (1) the distribution of the different versions of treatment (E_1 _vs. E_2_) -- a characteristic of the particular operationalization of the "treatment" in this particular study, and (2) the proportion of individuals who have different potential outcomes for E_1 _and E_2 _-- a characteristic of this population.

A Sufficient Component Cause model for this scenario is shown at the top of Figure 2. For some individuals, those with only the causal partner depicted by U_1_, bicycle riding would lead to weight loss only if they rode on the flats (E_1_) (because, for example, they would go more quickly and therefore expend more energy riding on the flats). Other individuals, those with only the causal partner depicted by U_2_, would lose weight only if they rode on the hills (E_2_) (because they have to work hard to climb the hill). Some individuals, those with U_1 _and U_2_, would lose weight either way (because they would ride quickly on the flats and work hard on the hills).

We imagined randomizing each individual to bicycle riding, but didn't specify the type of riding to which they were assigned. Each individual is exposed to only one version of bicycle riding, but we don't know which version because we didn't "represent" it in our study. In fact, because we don't represent the version of exposure, we don't even know the proportion of individuals who received the different versions of the exposure. These unrepresented versions of exposure would lead to unstable potential outcomes for some individuals, and therefore an undefined causal effect. For some individuals a comparison between their potential outcomes under the exposed and unexposed condition (whether it is 1 or 0), will depend on which version of treatment they receive.

The table in Figure 2 shows the potential outcomes under each version of exposure. In any particular randomization, individuals assigned to E would either get E_1_, or E_2_. The causal effect of the exposure would either be a comparison between E_1 _and Ê or E_2 _and Ê depending on which version of exposure they received. Rows in grey would not arise under monotonicity (e.g., if the exposure is only causal or neutral but never preventive compared with the no exposure condition).

Despite the difference in the versions of treatment, the potential outcomes of some individuals in the population will be stable: the doomed [those who have (X) or (U_1_, U_2 _and W)], the E_1 _and E_2 _causal (those who have U_1 _and U_2 _only), and the immune (those who have none of the component causes, U_1_, U_2_, W or X). Unstable individuals are those who do not have × and have either U_1 _or U_2_, but not both. As an example, consider individuals in row 4 (i.e., those who have U_1 _only). The exposure would have a causal effect for these individuals if they got E_1 _(i.e., the difference in their potential outcomes under this version of E vs. Ê is 1) but not if they got E_2_. If they got E_2 _the difference in their potential outcomes under this version of E vs. Ê is 0. What is the causal effect of E for these individuals? We can't say without specifying the version of treatment they received.

It should be noted that under monotonicity, anyone who is doomed regarding E_1 _is also doomed regarding E_2 _since once a monotonicity assumption is invoked, only individuals with × are doomed and they would be doomed regardless of the version of E to which they were exposed. Further, SUTVA is violated by different versions of treatment only if there are individuals in the population who would have a different potential outcome if exposed to the different versions of treatment (i.e., have only U_1 _or U_2 _but not both). If all individuals would respond the same way to both versions of treatment, this aspect of SUTVA would not be violated [5].

Note that in the example we used, the potential outcome of all individuals was stable under non-exposure since there was only one version of non-exposure. One could imagine more complex scenarios where the causal effect of interest is the difference between two active treatments, each of which could have unrepresented versions [5]. Nonetheless, in many epidemiologic contexts, where the causal effect is defined in terms of the presence or absence of E, only the potential outcomes under E would be affected.

This first aspect of SUTVA is violated to some extent in all studies [9,19]. The constructs that we measure or manipulate are never exactly what we intend. For example, in an RCT context, SUTVA is violated by non-compliance [5]. In an RCT we estimate causal effects of the treatment with the full recognition that there is some slippage between the causal effect that we often want (the effect of the active treatment) and the causal effect that we get (the effect of treatment assignment). Similarly, in an observational study, the exposures that individuals experience and that we measure are never exactly the same. To the extent that these different exposure experiences create different effects for some individuals, the causal effect that is estimated will not be very precise.

One solution to violations of this aspect of SUTVA is to represent the unrepresented versions of treatment. In our bike riding example, one could have two active treatments -- bike riding on hills and bike riding on flats -- instead of one. The more narrowly the exposure is defined, the less likely this aspect of SUTVA will be violated. However, there is a tension between the goal of defining an exposure narrowly enough to avoid SUTVA violations and defining a construct broadly enough that the effects can be generalized beyond the particular operationalization of the exposure in a study [9,20]. More sophisticated methods for estimating causal effects under multiple versions of treatment also depend on the representation of the previously unrepresented versions [21].

### When SUTVA is violated by interference between units, as described below, the situation is more complex

#### Violation of SUTVA through interference between units

The second aspect of SUTVA could be violated if some individuals were influenced by the exposure assignment of other individuals. For example, an individual randomized to no bike riding may notice that those who were assigned to the riding group seemed happier. In response, they began their own exercise regiment, (e.g., they started running), which leads to weight loss. Or individuals noticed that those in the bicycle group were losing weight and looked really good so in response they changed their diet. We refer to the people whose exposure status influences the outcomes of other people as "influential others". What makes others "influential" could be a characteristic of these individuals (e.g., they are powerful), their number (e.g., the proportion of people who are assigned to exposure) or the relationships among individuals (e.g., members of a social network). These "influential others" could affect an individual's potential outcome either independent of or dependent on the individuals' own exposure assignment. Both of these situations could lead to a SUTVA violation as explained below.

It should be noted that although everyone is "exposed" to these influential others (in the sense that they are in the same population) not everyone is affected by them. Influential others only have an effect on individuals with the necessary causal partners. These causal partners could, but need not, include contact between the influential others and the individual.

**Figure 3:**
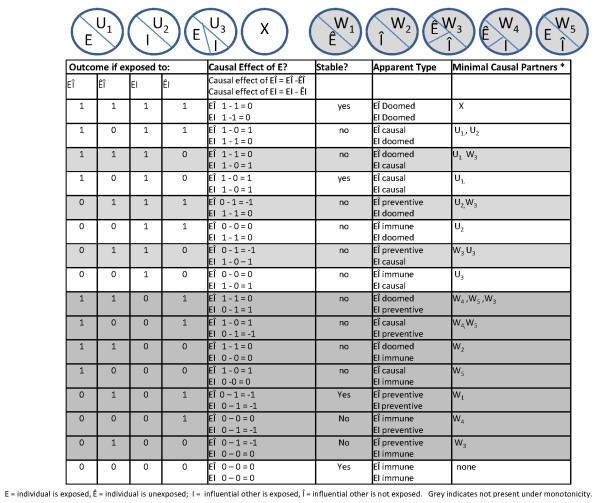
**SUTVA violations from interference between units**.

In this violation of SUTVA, although a single exposure was randomly assigned, the exposure essentially has two versions: one version when the individual receives the exposure in the presence of exposed influential others and another version when the individual receives the exposure in the presence of unexposed influential others. A Sufficient Component Cause model underlying this scenario is shown at the top of Figure 3. An individual's own exposure condition is indicated as E if exposed and Ê if not exposed. The exposure condition of influential others is indicated as I if the influential others are exposed and as Î if the influential others are unexposed.

Some individuals would lose weight if they are assigned to bicycle riding regardless of the exposure assignment of influential others -- they have U_1_, the causal partners of E_. _Some individuals would lose weight if influential others are assigned to bicycle riding regardless of their own exposure assignment -- they have the causal partners of I, U_2_. Other individuals may lose weight only if both they and influential others are assigned to the exposure group; they have U_3_, the causal partners of E and I.

For some individuals, those with W_1_, the exposure may be protective (e.g., they may lose weight only if they don't ride, perhaps because they over-eat when they ride). Likewise, the exposure of influential others may negatively affect the weight loss of some individuals, those with W_2_, if for example they are rebellious and do not want to exercise if others are doing so. Protective effects are indicated by the sufficient causes in grey and the rows in grey. If we invoke a monotonicity assumption (e.g., if we assume that neither the individual's own exposure, E, nor the exposure of influence others I, is ever preventive), only the white rows would be relevant. In many situations, such an assumption would be justified. We begin with this assumption and briefly discuss the implications of loosening this assumption later.

An individual may be randomized to exposure or not, when influential others are also exposed; the causal contrast depicting this effect is EI vs. ÊI. An individual may also be randomized to exposure or not when influential others are not assigned to the exposure group; EÎ vs. ÊÎ. These sets of potential outcomes are shown in the table in Figure 3. For some individuals, the potential outcomes are stable; whether their contrast is EI vs. ÊI or EÎ vs. ÊÎ, the causal effect of the exposure is the same. These individuals are the doomed, who lose weight regardless of their assignment or the assignment of anyone else (individuals who have X); those who are causal regardless of the assignment of others (individuals who have U_1 _only)_, _and those who are immune (individuals who do not have X, U_1 _or U_2_).

Three types of individuals have unstable potential outcomes: 1) Individuals who are susceptible both to their own exposure assignment as well as the assignment of influential others -- those with U_1 _and U_2 _only. Their causal effect is 1 if influential others are not exposed, but 0 if influential others are exposed; 2) Individuals who are susceptible to exposure only if influential others are also exposed -- those with U_3_. Their causal effect is 0 if influential others are not exposed but 1 if influential others are exposed; and 3) Individuals who are susceptible to the exposure of influential others, regardless of their own exposure -- individuals with U_2 _only. While this group has unstable potential outcomes (they are immune for EÎ vs. ÊÎ and doomed for EI vs. ÊI), their causal effect is stable. The exposure would have no causal effect for them in either scenario, but in one instance they lose weight and in the other they do not.

In any particular trial of this exposure, an individual is assigned to exposure or non-exposure, and influential others are assigned to exposure or non-exposure (i.e., EÎ vs. ÊÎ or EI vs. ÊI). The proportion of types represented in the population would then depend on the proportion of individuals in the population who were randomized to each exposure condition, the proportion of types in the population, and the distribution of influential others randomized to each exposure condition in the population.

In the example we used, "influential others" are characterized as individuals who would affect anyone with the appropriate causal partners. This would happen, for example, if influential others were simply older individuals, powerful individuals or a particular proportion of individuals. The situation can become far more complex when who is "influential" is dependent on social network relationships. So for example, if friendship links defined influential others, SUTVA would be violated when individuals with such links are included in the same study. These situations can also be conceptualized as causal partners of the exposure but the causal partners would need to include different sufficient causes for different influential others.

In addition, when the monotonicity assumption of no preventive effects is not reasonable, the number of situations where individuals will have unstable potential outcomes is substantially increased as indicated by the grey rows in Figure 3. One can easily imagine some scenarios where the exposure of influential others could have an adverse effect on other exposed individuals. For example, an exposure may lose its appeal when it becomes prevalent (e.g., the effectiveness of a virginity pledge [22]).

Like the first aspect of SUTVA, this second aspect of SUTVA (sometimes referred to as a "no interference between units assumption") [6,15] is also violated in many studies [5,9]. Infectious diseases provide a classic example, where the exposure of some individuals is the source of disease in others. Similar processes shape human behaviors and actions. To the extent that we study exposures which are at least partially under an individual's control or shaped by social processes, this aspect of SUTVA will also be violated [19].

Various solutions to violations of this aspect of SUTVA have been proposed. All require either the elimination of the interference between units through a narrowing of the causal question [23] or a recognition and representation of the interference [8]. This parallels solutions to the problems of unrepresented versions of treatment. For SUTVA to hold, the different versions of the exposure and the interference between units need to be eliminated or represented (e.g., [24-27]) For example, if spouses both appear in a study and for some individuals the effect of the exposure depends on the spouse's exposure, a solution would be to consider four rather than two exposure conditions - an individual can be exposed or not when the spouse is exposed and the individual can be exposed or not when the spouse is not exposed [28]. Another approach is to separate the causal questions to represent the different versions of treatment in separate causal analyses [23,29] distinguishing between the direct effects of the exposure and indirect effects that are a consequence of interference between units.

## Conclusion

### SUTVA for causal generalization

SUTVA is clearly a requirement for estimating the precise causal effect defined from a potential outcomes frame. [5,9,10][12]. Rooted in manipulation theories of causation, the goal of a study is to estimate the effect of manipulating the exposure. If SUTVA is violated, one cannot consistently predict the effect of manipulating the exposure on the outcome and thus the causal effect is not unitary or stable. The correspondence between the study results and an intervention effect will depend heavily on the extent to which the distribution of the versions of treatment in the study matches the distribution of the versions in the intervention [12].

Continuing our example, suppose a large study effect for bicycle riding was found, but it was due to the fact that most of the rides took place on hills. Suppose further that the researchers, unaware of this unrepresented version of treatment, mounted an intervention where all the rides took place on the flats. The intervention will not replicate the study effect. Similarly, if in the study the exposure's effect were contingent on the assignment of influential others and the intervention did not include influential others, the study and intervention effects could be quite different. Exchangeability and SUTVA are both necessary assumptions for the estimation of this precise causal effect, the effect that changing the exposure would have in this population. That is, SUTVA is required for transportability of the study's exposure effect not only to different populations, [12] but even to the effect of an actual intervention in the study population [30,31].

However, examining SUTVA from the context of a Sufficient Component Cause model, a model that highlights the contingency of causal effects, implies a critical distinction between exchangeability and SUTVA in their respective roles in causal inference. Exchangeability is a requirement for the internal validity of the results; without exchangeability, association does not indicate causation. By causation, we mean the difference between what actually happened and what would have happened except that the exposure was absent and all else were equal [16]. Without exchangeability, even the interpretation of the association between the exposure and disease as an actual local effect (that is the effect the exposure actually had in the study) is suspect. SUTVA, on the other hand, is not required for identifying the actual causes of the outcome in the actual extant circumstances.

Rather, SUTVA is required for transportability -- for generalization of the causal effect outside of the particular conditions of the study at hand [20,32]. The first aspect of SUTVA, no unrepresented versions of treatment, is necessary to generalize the causal effect of the exposure beyond its particular operationalization in the study - consideration of construct validity. The second aspect of SUTVA is required for generalization to settings that differ from the one under study -- consideration of external validity. Shadish, Cook and Campbell's [20] validity scheme helps to articulate this distinction. In their schema, they distinguish between causal description and causal generalization. Causal description (what epidemiologists would refer to as internal validity) entails the identification of a causal relationship between two variables as they were manipulated or measured within a particular context [31]. Causal explanation entails an understanding of "the mechanisms through which (construct validity) and the conditions under which (external validity) that causal relationship holds" p.9.

In examining Figure 2, the causal effect of the exposure as operationalized in the study is identifiable (i.e. the exchangeability assumption is met and the study is internally valid). This operationalization represents the average of the effects of the versions of treatment as they are distributed in the study in this particular population. It is a true causal effect, but one that may not be replicable without further analysis of the effects of the particular operationalization of the exposure in the study. That is, without representing the unrepresented versions of treatment, the first part of SUTVA is violated and the precise meaning of the exposure construct is unclear. To continue our example, the causal effect identified in the study would be the effect of bicycle riding across conditions of hills and flats as they arose in this particular population at this particular moment in time. Just as the heterogeneity of effects across individuals limits all studies to the identification of the average causal effect across individuals in a population, so too the heterogeneity of the versions of treatment yields an average causal effect across the different, unrepresented versions of treatment in the study. It is a true causal effect, not just an association, but not a causal effect whose magnitude will necessarily generalize to other operationalizations of the exposure.

Similarly, the effect of the treatment assignment of some individuals on the outcomes of others, renders the causal effect identified as the actual causal effect given the particular context in which a particular group of individuals is exposed or given the context of a particular distribution of influential others. This can be seen in Figure 3, where the effect of influential others modifies the effect of the exposure of the individual. This effect modification, the distribution of the causal partners, is what makes a causal effect context dependent. For the causal effect to generalize from the study results to an intervention effect, the second aspect of SUTVA (external validity in Shadish, Cook and Campbell's scheme) must hold.

SUTVA violations contrast sharply with violations of the exchangeability assumption. Without exchangeability, both the estimated causal effect defined in terms of the effect the exposure actually had in the study context and the estimated effect it would have in the future would be misleading. To the extent that non-exchangeability was responsible for the exposure-disease association, the association did not reflect a causal effect of the exposure on the outcome.

It may be that SUTVA has not been as prominent in epidemiology as exchangeability because much of epidemiology has been concerned with questions about the causes of effects -- the identification of factors that, in at least some contexts, cause an outcome. For this task, exchangeability is dominant. The potential outcomes frame leads to a different type of causal inquiry, questions about the effects of causes, effects of the manipulation of the exposure. It may be that the causal effects we estimate in our studies, even when SUTVA holds, should be more modestly considered as a task of causal identification rather than the effect of causal manipulation.

The SCC model allows us to see the intimate relationship between interaction and SUTVA violations. Because we assume that interaction is ubiquitous, we acknowledge that the effects we estimate in our studies are the average of different causal effects. We do this with the recognition that the average causal effect can be very misleading for some purposes particularly if there is qualitative interaction -- that is if an exposure can be causal in the presence of some causal partners and protective in others. In future studies we try and identify some of the causal partners of the exposure that we think account for dramatic differences in the exposure's effect.

Examining SUTVA violations through an SCC lens, reinforces the analogy with interaction - the causal effect in any study represents the average of the effect of the different versions of treatment that are unarticulated and unmeasured.

We think that the implications of these different approaches to framing causal questions deserve more attention [19]. We also think that a fuller consideration of the relationship between Shadish, Cook and Campbell's schema and the potential outcomes approach to causation in epidemiology, could be beneficial [32-34].

In this paper, we presented simple Sufficient Component Cause models depicting SUTVA violations to describe the basic elements of this assumption. A fuller explication will need to take into account preventive effects and the consequences of SUTVA violations for the viability of the exchangeability assumption.

## Abbreviations

SUTVA: Stable Unit Treatment Value Assumption; SCC: Sufficient Component Cause.

## Competing interests

The authors declare that they have no competing interests.

## Authors' contributions

SS took the lead on researching and writing this article which arose from ongoing research and discussion with and critical rewrites from NG and UC. All authors read and approved the final manuscript.
